# Supplementary material for: Content-rich biological network constructed by mining PubMed abstracts
Source: BMC Bioinformatics. 2004 Oct 8;5:147. doi: 10.1186/1471-2105-5-147 (PMC528731; doi:10.1186/1471-2105-5-147)
Supplement: Additional File 2 — The original results of the above study (non-essential files are deleted to keep the file size under the limit set by BMC bioinformatics). [file 1471-2105-5-147-S2.bz2 › chilibotAdditionalFile2/dip40/339ID1280226E4221/html/TNFSF5_TNFRSF5.html]

 


 **TNFSF5** and **TNFRSF5** 
  
Found 2289 abstracts in PubMed, retrieved 40.  
 

 What does Google say? 
 PDF only 
| .edu only 

---

**Interactive relationship** (e.g. stimulation, inhibition, etc)

**Neutral relationship**- Proangiogenic Function of CD40  [ **TNFRSF5** ]  Ligand  [ **TNFSF5** ]  CD40  [ **TNFRSF5** ]  Interactions.  Ref: 12874247 J Immunol, 2003
- These patients presented opportunistic infections as observed in X linked HIGM, suggesting that the CD40  [ **TNFRSF5** ]  CD40  [ **TNFRSF5** ]  ligand  [ **TNFSF5** ]  interaction is important for promoting T cell mediated immunity.  Ref: 12893749 BloodBlood, 2003
- Interactions between CD40  [ **TNFRSF5** ]  ligand  [ **TNFSF5** ]  CD40L  [ **TNFSF5** ]  and CD40  [ **TNFRSF5** ]  have been shown to have pluripotent functions in inflammation, including the production of cytokines, chemokines, as well as the angiogenesis factor, vascular endothelial growth factor VEGF, by endothelial cells.  Ref: 12874247 J Immunol, 2003
- CONCLUSION CD40  [ **TNFRSF5** ]  CD40  [ **TNFRSF5** ]  ligand  [ **TNFSF5** ]  interaction induced a robust stimulation of the DAG PKC pathway and calcium mobilization from intracellular pool in PBMC.  Ref: 12852836 Acta Pharmacol Sin, 2003
- The CD40  [ **TNFRSF5** ]  CD40  [ **TNFRSF5** ]  ligand  [ **TNFSF5** ]  CD40L  [ **TNFSF5** ]  interaction is one of the most important receptor ligand interactions that occurs during a T dependent immune response.  Ref: 12848855 Immunol Cell Biol, 2003
- Although the interaction seems to play a crucial role in asthmatic inflammation, previous studies investigating the role of the CD40  [ **TNFRSF5** ]  and CD154  [ **TNFSF5** ]  interaction in experimental animal models of asthma are complicated due to multistep reactions in developing asthma.  Ref: 12823274 Clin Exp Immunol< MedlineTA>Clin Exp Immunol, 2003
- and 2 unopposed activation of Th2 cytokine producing CD4 T cells resulting in B cell hyperreactivity and dysregulated immunoglobulin synthesis through Th2 cytokines and heightened CD40  [ **TNFRSF5** ]  ligand  [ **TNFSF5** ]  CD40  [ **TNFRSF5** ]  interactions.  Ref: 12820736 Ann Thorac Surg, 2003
- The interaction between CD40  [ **TNFRSF5** ]  and its ligand CD154  [ **TNFSF5** ]  is crucial for IL 12 production and effective humoral immunity such as IgE production.  Ref: 12823274 Clin Exp Immunol< MedlineTA>Clin Exp Immunol, 2003
- Effect of CD40  [ **TNFRSF5** ]  CD40  [ **TNFRSF5** ]  ligand  [ **TNFSF5** ]  interaction on diacylglycerol protein kinase C signal transduction pathway and intracellular calcium in cultured human monocytes.  Ref: 12852836 Acta Pharmacol Sin, 2003
- HBcAg dependent IL 18 levels were abrogated by inhibition of Caspase 1, but not by blockade of CD40  [ **TNFRSF5** ]  CD154  [ **TNFSF5** ]  interaction.  Ref: 12858406 J Med Virol, 2003

**Non-interactive relationship** (e.g. studied together, co-existance, homology, etc.)

- Blockade of the CD40  [ **TNFRSF5** ]  CD154  [ **TNFSF5** ]  pathway enhances T cell depleted allogeneic bone marrow engraftment under nonmyeloablative and irradiation free conditioning therapy.  Ref: 12865813 Transplantation, 2003
- BACKGROUND CD40  [ **TNFRSF5** ]  CD40  [ **TNFRSF5** ]  ligand  [ **TNFSF5** ]  CD40L  [ **TNFSF5** ]  signaling is a potent activator of endothelial cells ECs and promoter of atherosclerosis.  Ref: 12885753 CirculationCirculation, 2003
- BACKGROUND CD40  [ **TNFRSF5** ] , a member of the tumor necrosis factor  [ **TNFSF5** ]  receptor superfamily, is capable of mediating the induction of apoptosis in tumors including sarcomas.  Ref: 12820428 Anticancer Res< MedlineTA>Anticancer Res, 2003
- Furthermore, resistin upregulates adhesion molecules and chemokines and downregulates TRAF 3, an inhibitor of CD40  [ **TNFRSF5** ]  ligand  [ **TNFSF5** ]  signaling.  Ref: 12874180 CirculationCirculation, 2003
- Stimulation via CD40  [ **TNFRSF5** ]  CD40L  [ **TNFSF5** ]  resulted in increased viability, as did stimulation with IL 4.  Ref: 12860016 Leuk Res, 2003
- Induction of an Ig class switch in nude mice was related to the expression of IFN gamma and CD40  [ **TNFRSF5** ]  ligand  [ **TNFSF5** ]  in the draining lymph nodes.  Ref: 12847220 J Immunol, 2003
- CONCLUSIONS A20 works at multiple levels to protect ECs from CD40  [ **TNFRSF5** ]  CD40L  [ **TNFSF5** ]  mediated activation and apoptosis.  Ref: 12885753 CirculationCirculation, 2003
- Granulocyte monocyte colony stimulating factor, interleukin 4, and CD40  [ **TNFRSF5** ]  ligand  [ **TNFSF5** ]  in combination are capable of yielding dendritic cells from at least some cases of acute lymphocytic leukemia.  Ref: 12850495 Blood Cells Mol Dis< MedlineTA>Blood Cells Mol Dis, 2003
- The cells of chronic lymphocytic leukemia were treated in vitro with CD40  [ **TNFRSF5** ]  ligand  [ **TNFSF5** ]  or IL 4 to explore their effects on survival and sensitivity to apoptosis induced by Fluda.  Ref: 12860016 Leuk Res, 2003
- RESULTS IKK 2 was not required for lipopolysaccharide LPS induced NF kappaB activation or TNFalpha, IL 6, or IL 8 production in macrophages, but was essential for this process in response to CD40  [ **TNFRSF5** ]  ligand  [ **TNFSF5** ] , TNFalpha, and IL 1.  Ref: 12847684 Arthritis Rheum, 2003
- The activity of PKC and Ca2 i induced by CD40  [ **TNFRSF5** ]  ligand  [ **TNFSF5** ]  CD40L  [ **TNFSF5** ]  in PBMC were measured by its ability to transfer phosphate from gamma 32P ATP to lysine rich histone and flow cytometric analysis loading with the Ca2 dye Fluo 3 Am, respectively.  Ref: 12852836 Acta Pharmacol Sin, 2003
- Altogether, these data show that in contrast to CD40  [ **TNFRSF5** ]  ligand  [ **TNFSF5** ] , which induced LC maturation even in presence of TGF beta1, nonspecific proinflammatory factors such as TNF alpha, IL 1 or LPS, essentially induced immature LC generation, and little cell activation in the presence of TGF beta1.  Ref: 12884872 Eur J Immunol, 2003
- CD154  [ **TNFSF5** ]  CD40  [ **TNFRSF5** ]  ligand  [ **TNFSF5** ]  has a wide variety of pleiotropic effects throughout the immune system and is critical to both cellular and humoral immunity.  Ref: 12857968 Immunol Res, 2003
- By examining surface expression of numerous candidate molecules tumor necrosis factor  [ **TNFSF5** ]  receptor TNFR 1, TNFR2, CD36, CD40  [ **TNFRSF5** ] , CD47, CD80, CD86, PD L1, CD95 ligand, and type I interferon receptor, we report that IPE cells uniquely express on their surface the costimulatory molecule CD86.  Ref: 12835481 J Exp Med, 2003
- Methods Keratinocytes were grown on fibroblasts transfected with the CD40  [ **TNFRSF5** ]  ligand  [ **TNFSF5** ]  CD40L  [ **TNFSF5** ]  to investigate the formation of epidermal sheets in culture under the influence of the CD40L  [ **TNFSF5** ] .  Ref: 12828736 Br J Dermatol, 2003
- Treatment with the DC maturation factor trimeric CD40  [ **TNFRSF5** ]  ligand  [ **TNFSF5** ]  CD40  [ **TNFRSF5** ]  L  [ **TNFSF5** ]  either alone or in combination with fl3 L neither prevented loss of DC class II antigens nor disease relapse.  Ref: 12827302 Cancer Immunol Immunother< MedlineTA>Cancer Immunol Immunother, 2003
- Moreover, their expression of class II, CD40  [ **TNFRSF5** ] , B7 1 and B7 2 molecules, and the mRNA for potential angiogenic factors, such as vascular endothelial growth factor, basic fibroblast growth factor, and tumor necrosis factor  [ **TNFSF5** ]  alpha, was also observed.  Ref: 12832439 J Leukoc Biol< MedlineTA>J Leukoc Biol, 2003
- Allergic diseases have been a model study for Th2 cells polarization and it is well known that the synthesis of the human IgE results from collaboration between Th2 and B cells through the cytokines such as IL 4 and IL 13 and surface molecules CD40  [ **TNFRSF5** ]  CD40L  [ **TNFSF5** ] .  Ref: 12825491 Rev Alerg Mex< MedlineTA>Rev Alerg Mex, 2003
- It is not know whether donor T cells are essential for bone marrow engraftment and whether blocking the CD40  [ **TNFRSF5** ]  CD154  [ **TNFSF5** ]  pathway promotes allogeneic TDBM engraftment under nonmyeloablative and irradiation free fludarabine phosphate and cyclophosphamide conditioning therapy.METHODS Using fully major histocompatibility complex MHC matched mouse models, we investigated whether donor T cells are essential for bone marrow engraftment under fludarabine phosphate and cyclophosphamide conditioning therapy.  Ref: 12865813 Transplantation, 2003
- Why do B cells produce CD40  [ **TNFRSF5** ]  ligand  [ **TNFSF5** ]  ? < ArticleTitle>.  Ref: 12848855 Immunol Cell Biol, 2003
- Our findings suggest that although the potential of myeloid DC precursors to achieve full maturation is preserved in subjects with late stage HIV 1 infection, in vivo maturation of myeloid DCs was impaired in these subjects, which may be due to decreased potential of CD40  [ **TNFRSF5** ]  ligand  [ **TNFSF5** ]  expression on CD4 T cells.  Ref: 12869829 J Acquir Immune Defic SyndrJ Acquir Immune Defic Syndr, 2003
- AIM To investigate whether CD40  [ **TNFRSF5** ]  CD40  [ **TNFRSF5** ]  ligand  [ **TNFSF5** ]  interaction can activate diacylglycerol DAG protein kinase C PKC signaling pathway and intracellular free calcium Ca2 i in cultured human peripheral blood monocytes PBMC.  Ref: 12852836 Acta Pharmacol Sin, 2003
- Efficient VLP cross presentation by Langerhans cells with costimulation can be achieved by addition of CD40  [ **TNFRSF5** ]  ligand  [ **TNFSF5** ] .  Ref: 12839929 Cancer Res, 2003
- When added to GM CSF, IL 4 and TGF beta1 containing cultures, LPS or IL 1beta also induced significant numbers of Langerin CD83 immature cells displaying a low allostimulatory activity, while CD40  [ **TNFRSF5** ]  ligand  [ **TNFSF5** ]  largely promoted highly allostimulatory Langerin CD83 cells.  Ref: 12884872 Eur J Immunol, 2003
- The effects of resistin on expression of vascular cell adhesion molecule VCAM 1 and monocyte chemoattractant chemokine MCP 1 were studied in addition to CD40  [ **TNFRSF5** ]  receptor, CD40  [ **TNFRSF5** ]  ligand  [ **TNFSF5** ]  induced MCP 1 expression, and tumor necrosis factor  [ **TNFSF5** ]  receptor associated factor 3 TRAF3, an inhibitor of CD40  [ **TNFRSF5** ]  signaling.  Ref: 12874180 CirculationCirculation, 2003
- We also determined whether the barrier of allogeneic TDBM could be overcome by blocking the CD40  [ **TNFRSF5** ]  CD154  [ **TNFSF5** ]  pathway.  Ref: 12865813 Transplantation, 2003
- Furthermore, both hCD100 Fc and anti human CD72 agonistic mAb induce the production of the pro inflammatory cytokines tumor necrosis factor  [ **TNFSF5** ]  alpha, IL 6 and IL 8, even in the absence of anti CD40  [ **TNFRSF5** ]  mAb.  Ref: 12882840 Int ImmunolInt Immunol, 2003
- In this study, we investigate whether A20 a gene we have shown to be antiinflammatory and antiapoptotic in ECs can protect from CD40  [ **TNFRSF5** ]  CD40L  [ **TNFSF5** ]  mediated EC activation.  Ref: 12885753 CirculationCirculation, 2003
- A20 Protects From CD40  [ **TNFRSF5** ]  CD40  [ **TNFRSF5** ]  Ligand  [ **TNFSF5** ]  Mediated Endothelial Cell Activation and Apoptosis.  Ref: 12885753 CirculationCirculation, 2003
- X linked hyper IgM syndrome HIGM1 MIM musical sharp 308230, is a severe primary immunodeficiency caused by mutations in the gene coding for CD40  [ **TNFRSF5** ]  ligand  [ **TNFSF5** ]  CD40L  [ **TNFSF5** ]  or CD154  [ **TNFSF5** ] , a member of the tumour necrosis factor TNF superfamily.  Ref: 12823286 Clin Exp Immunol, 2003
- Both interleukin 4 and CD40  [ **TNFRSF5** ]  ligand  [ **TNFSF5** ]  are important for optimal dendritic cell differentiation and maturation.  Ref: 12850495 Blood Cells Mol Dis< MedlineTA>Blood Cells Mol Dis, 2003
- Activation of CD40  [ **TNFRSF5** ]  by CD154  [ **TNFSF5** ]  induces antigen presenting cells APC to express immune costimulatory molecules, thereby enhancing their APC activity.  Ref: 12874017 Cancer Res, 2003
- costimulatory molecule blockade must be directed against both the B7 CD28 and CD40  [ **TNFRSF5** ]  CD40L  [ **TNFSF5** ]  pathways.  Ref: 12829914 Transplantation, 2003
- Donor chimerism was established by TDBMT induced donor specific tolerance in vivo and in vitro.CONCLUSION Donor T cells facilitate bone marrow engraftment under nonmyeloablative and irradiation free conditioning therapy, and the blocking the CD40  [ **TNFRSF5** ]  CD154  [ **TNFSF5** ]  pathway can replace donor T cells to promote TDBM engraftment.  Ref: 12865813 Transplantation, 2003
- The results showed that the capacity of circulating DCs was significantly impaired in subjects with CD4 T cell counts of < ; 200 μL, correlated with the potential of CD40  [ **TNFRSF5** ]  ligand  [ **TNFSF5** ]  expression on CD4 T cells R = 0.84.  Ref: 12869829 J Acquir Immune Defic SyndrJ Acquir Immune Defic Syndr, 2003
- increased CD40  [ **TNFRSF5** ]  ligand  [ **TNFSF5** ]  induced MCP 1 production.  Ref: 12874180 CirculationCirculation, 2003
- The molecular defects that have so far been associated with this syndrome which affect the CD40  [ **TNFRSF5** ]  ligand  [ **TNFSF5** ]  in HIGM type 1 HIGM1, CD40  [ **TNFRSF5** ]  in HIGM3, and activation induced cytidine deaminase AID in HIGM2 do not account for all cases.  Ref: 12840068 J Clin Invest< MedlineTA>J Clin Invest, 2003
- Anti CD40  [ **TNFRSF5** ]  monoclone antibody 10 mg L significantly suppressed CD40L  [ **TNFSF5** ]  induced DAG PKC signal transduction pathway activation and Ca2 i changes in PBMC.  Ref: 12852836 Acta Pharmacol Sin, 2003
- When challenged, CD4 T cells from FoxP3 Tg mice display reduced up regulation of CD40  [ **TNFRSF5** ]  ligand  [ **TNFSF5** ]  and fewer IFN gamma producing cells.  Ref: 12874208 J Immunol, 2003
- The altered responsiveness of activated memory B cells to CD40L  [ **TNFSF5** ]  and BAFF correlated with changes in surface phenotype such that expression of CD40  [ **TNFRSF5** ]  and BAFF R were reduced on ISCs while BCMA was induced.  Ref: 12865416 J Clin Invest, 2003
